# FGFR families: biological functions and therapeutic interventions in tumors

**DOI:** 10.1002/mco2.367

**Published:** 2023-09-23

**Authors:** Qing Liu, Jiyu Huang, Weiwei Yan, Zhen Liu, Shu Liu, Weiyi Fang

**Affiliations:** ^1^ Cancer Center Integrated Hospital of Traditional Chinese Medicine Southern Medical University Guangzhou Guangdong China; ^2^ Key Laboratory of Protein Modification and Degradation Basic School of Guangzhou Medical University Guangzhou Guangdong China; ^3^ Department of Breast Surgery The Affiliated Hospital of Guizhou Medical University Guiyang Guizhou China

**Keywords:** fibroblast growth factor, fibroblast growth factor receptor, signaling pathway, tumor, tyrosine kinase inhibitors (TKIs)

## Abstract

There are five fibroblast growth factor receptors (FGFRs), namely, FGFR1–FGFR5. When FGFR binds to its ligand, namely, fibroblast growth factor (FGF), it dimerizes and autophosphorylates, thereby activating several key downstream pathways that play an important role in normal physiology, such as the Ras/Raf/mitogen‐activated protein kinase kinase/extracellular signal‐regulated kinase, phosphoinositide 3‐kinase (PI3K)/AKT, phospholipase C gamma/diacylglycerol/protein kinase c, and signal transducer and activator of transcription pathways. Furthermore, as an oncogene, FGFR genetic alterations were found in 7.1% of tumors, and these alterations include gene amplification, gene mutations, gene fusions or rearrangements. Therefore, FGFR amplification, mutations, rearrangements, or fusions are considered as potential biomarkers of FGFR therapeutic response for tyrosine kinase inhibitors (TKIs). However, it is worth noting that with increased use, resistance to **TKIs** inevitably develops, such as the well‐known gatekeeper mutations. Thus, overcoming the development of drug resistance becomes a serious problem. This review mainly outlines the FGFR family functions, related pathways, and therapeutic agents in tumors with the aim of obtaining better outcomes for cancer patients with FGFR changes. The information provided in this review may provide additional therapeutic ideas for tumor patients with FGFR abnormalities.

## Graphical abstract

FGFR amplification, mutations, fusions, or rearrangements can activate many pathways that lead to tumor development, and FGFR inhibitory drugs, especially TKIs, exert antitumor effects by blocking these pathways, but at the same time, secondary drug resistance inevitably arises in some patients, as overcoming drug resistance becomes a challenge that we must overcome.

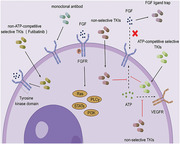

## 1 INTRODUCTION

A tumor is formed by the abnormal proliferation of cells due to the loss of normal regulation of local tissue cells at the gene level under the action of various tumorigenic factors, such as viruses, pollution, tobacco, alcohol, and chemical carcinogens,^1^, ^2^, ^3^, ^4^, ^5^, ^6^, ^7^, ^8^, ^9^, ^10^, ^11^, ^12^, ^13^, ^14^, ^15^, ^16^, ^17^, ^18^, ^19^, ^20^, ^21^, ^22^, ^23^ which is attributed to the action of these factors in stimulating or inactivating key signaling pathways and genes, such as the phosphoinositide 3‐kinase (PI3K)/AKT, Wnt/β‐catenin, Ras/extracellular signal‐regulated kinase (ERK), nuclear factor kappa B (NF‐κB), phospholipase C gamma (PLCγ), sting, signal transducer and activator of transcription (STAT), fibroblast growth factor (FGF)/fibroblast growth factor receptor (FGFR), c‐Myc, p53, and so on.^24^, ^25^, ^26^, ^27^, ^28^, ^29^, ^30^, ^31^, ^32^, ^33^, ^34^, ^35^, ^36^, ^37^, ^38^, ^39^, ^40^, ^41^, ^42^ Among them, the FGF/FGFR pathway plays a significant role in maintaining normal physiological balance of the human body.

FGF is the key ligand of FGFR. Normally, when FGFR is triggered by FGF, a lot of pathways involved in many physiological activities, such as cell growth, cell migration, cell survival, angiogenesis, embryonic organ development, tissue repair, wound healing, and metabolism are activated. The event is caused by the auto‐phosphorylation of FGFR.^43^, ^44^, ^45^, ^46^, ^47^ The FGFR family includes five genes, namely, *FGFR1*, *FGFR2*, *FGFR3*, *FGFR4*, and *FGFR5 (FGFRL1)*. FGFR1–4 auto‐phosphorylated tyrosine residues bind and phosphorylate adaptor proteins, such as FGFR substrate 2 (FRS2). FRS2 consists of a phosphotyrosine‐binding domain and a C‐terminal tail with multiple binding sites for the SH2 domains of growth factor receptor‐bound 2 (GRB2) and Src homology phosphatase 2 (Shp2).^48^, ^49^ Phosphorylated FRS2 recruits GRB2 and son of sevenless (SOS) to activate Ras and its downstream mitogen‐activated protein kinase (MAPK) pathway.^50^ GRB2‐associated binding protein 1 (GAB1) binds to GRB2 and activates the AKT pathway. In addition to FRS2, PLCγ binds to the Y766 phosphorylated tyrosine residue of FGFR to activate the PLCγ/diacylglycerol/protein kinase C (PKC) pathway.^51^ These pathways are involved in many physiological processes. For example, FGF2/FGFR regulates cumulus expansion and oocyte meiosis in mouse, which may be achieved due to the activation of the c‐Mos/MAPK pathway.^52^ Under apoptotic stress, cells release FGF2, which promotes transcriptional upregulation of B‐cell lymphoma‐2 (BCL‐2) protein in a non‐cell‐autonomous manner through activation of the mitogen‐activated protein kinase kinase (MEK)/ERK axis, which protects neighboring cells from apoptotic damage.^53^ The FGFR1/Janus kinase 2 (JAK2)/STAT3 signaling pathway, which is enhanced by human b‐defensin‐3, promotes wound healing, angiogenesis, and fibroblast activation.^43^ FGF4 activates AMP‐activated protein kinase/Caspase 6 signal axis through FGFR4 to regulate liver stress response, reduce stem cell apoptosis and mitigate liver injury.^54^

Notably, in addition to the typical FGF/FGFR pathways mentioned above, there are also some atypical examples. For instance, in Xenopus embryos, FGFR is activated by family with sequence similarity 3 member B to activate downstream ERK signaling and promote posterior development, forming an anterior–posterior axial pattern.^55^ Furthermore, activation of ERK induces remodeling of cytoskeletal proteins, including F‐actin, embryonic calmodulin C‐cadherin, and the tight junction protein zonula occluden‐1, which is required for enhanced cellular junctions and tissue sclerosis during early embryogenesis. It is worth noting that, here, the FGFR1/ERK pathway is not FGF‐dependent due to mechanical stress.^56^ FGF/FGFR2 was shown to enhance the SHH–BMP4 signaling axis in the mouse, thereby promoting uroepithelial and mesenchymal development in the early ureter.^57^ In the liver, FGF15 acts on FGFR4, recruits and phosphorylates NF2, deregulates the inhibition of Hippo kinases Mst1/2 by Raf, which activates the Hippo pathway, downregulates the expression of bile acid synthase Cyp7a1, and limits bile acid synthesis.^58^

At the same time, abnormal regulation of these pathways is also involved in the development and progression of many diseases, especially tumors. For example, FGFR in oligodendrocytes downregulates brain‐derived neurotrophic factor/tropomyosin receptor kinase B signaling via the ERK/AKT pathway, which may be associated with the development of multiple sclerosis.^59^ Overexpression of high molecular weight FGF2 heterodimer (HMWFGF2), which activates Wnt signaling by targeting FGFR, is involved in the development of osteoarthrosis.^60^ In tumor cells, FGFR inhibits the JAK/STAT signaling pathway activated by T cell‐produced interferon gamma and then reduces the expression of its target genes, namely, beta‐2 microglobulin (B2M), C‐X‐C motif chemokine ligand 10 (CXCL10), and programmed death‐ligand 1 (PD‐L1), thereby mediating immune escape.^60^ In non‐small cell lung cancer (NSCLC), FGFR activates the MAPK/c‐Fos pathway to promote tumor cell proliferation, migration, and resistance to erlotinib.^61^ Similarly, FGFR promotes cholangiocarcinoma cell progression and resistance to gemcitabine through upregulation of AKT/mammalian target of rapamycin (mTOR) and STAT3 signaling.^62^ The AKT/SRY‐box 2 (SOX2) axis, mediated by FGFR2, also controls the stemness of pancreatic cancer.^63^

In this review, we will summarize the role of the FGFR family involved in human tumors, related regulatory signaling, and possible targeted interventions. The information offered in this review will help to highlight the role of FGFR proteins in tumor pathogenesis as well as the possibility of FGFR proteins as targets for tumor therapy.

## 2 OVERVIEW OF THE FGFR FAMILY

### 2.1 Major ligands of FGFR

FGFs are the main ligands of FGFR. The FGF family has 18 canonical isoforms in human, namely, FGF1 to FGF10 and FGF16 to FGF23, and their molecular weights range from 15 to 38 kDa. The FGF family can be divided into the following six subfamilies: FGF1 (FGF1 and FGF2); FGF4 (FGF4, FGF5, and FGF6); FGF7 (FGF3, FGF7, FGF10, and FGF22); FGF8 (FGF8, FGF17, and FGF18); FGF9 (FGF9, FGF16, and FGF20); and FGF19 (FGF19, FGF21, and FGF23). Of these, five subfamilies are paracrine subfamilies, and one subfamily (FGF19 subfamily) is an endocrine subfamily.^64^, ^65^, ^66^, ^67^ These FGFs act by binding, dimerizing, and activating the tyrosine kinase of the FGFR and its downstream pathways.^68^, ^69^ It is worth noting that paracrine subfamilies also bind to heparin or heparan sulfate (HS) proteoglycan, which does not transmit signals but acts as a cofactor to shorten the diffusion distance between FGF and its secretory cells as well as regulates the binding and signaling of FGF to its receptor (FGFR).^70^, ^71^, ^72^, ^73^ In addition, the binding of FGF to HS stabilizes growth factors, limits growth factor diffusion, and provides cells with growth factor reserves.^74^, ^75^, ^76^ The HS binding site on FGF consists of the β1‐β2 loop and a region composed of the β10 strand, β10‐β11 loop, β11 strand, and β11‐β12 loop.^69^

### 2.2 The structure of FGFR

The conventional FGFR has four different members, namely, FGFR1 to FGFR4, which consist of an extracellular domain consisting of three immunoglobulin‐like domains (D1, D2, and D3), a single transmembrane domain, and an intracellular domain containing a tyrosine kinase domain.^77^, ^78^ In particular, D1 and D2 are linked by a sequence of seven to eight amino acids rich in glutamate, aspartate, and serine, which is known as the acid box (AB). D1 and AB play a key role in the auto‐inhibition of FGFR.^79^, ^80^ D2 has an HS‐binding site, which consists of a conserved positively charged region.^81^ The D2‐D3 regions are critical for ligand binding and specificity.^82^ FGFR1 to FGFR3 are selectively spliced to produce b and c isoforms,^83^, ^84^, ^85^ and this splicing event modulates the specificity of FGFR ligand binding by altering the primary sequence of the critical ligand binding region in the C‐terminal half of D3.^86^, ^87^

**FIGURE 1:**
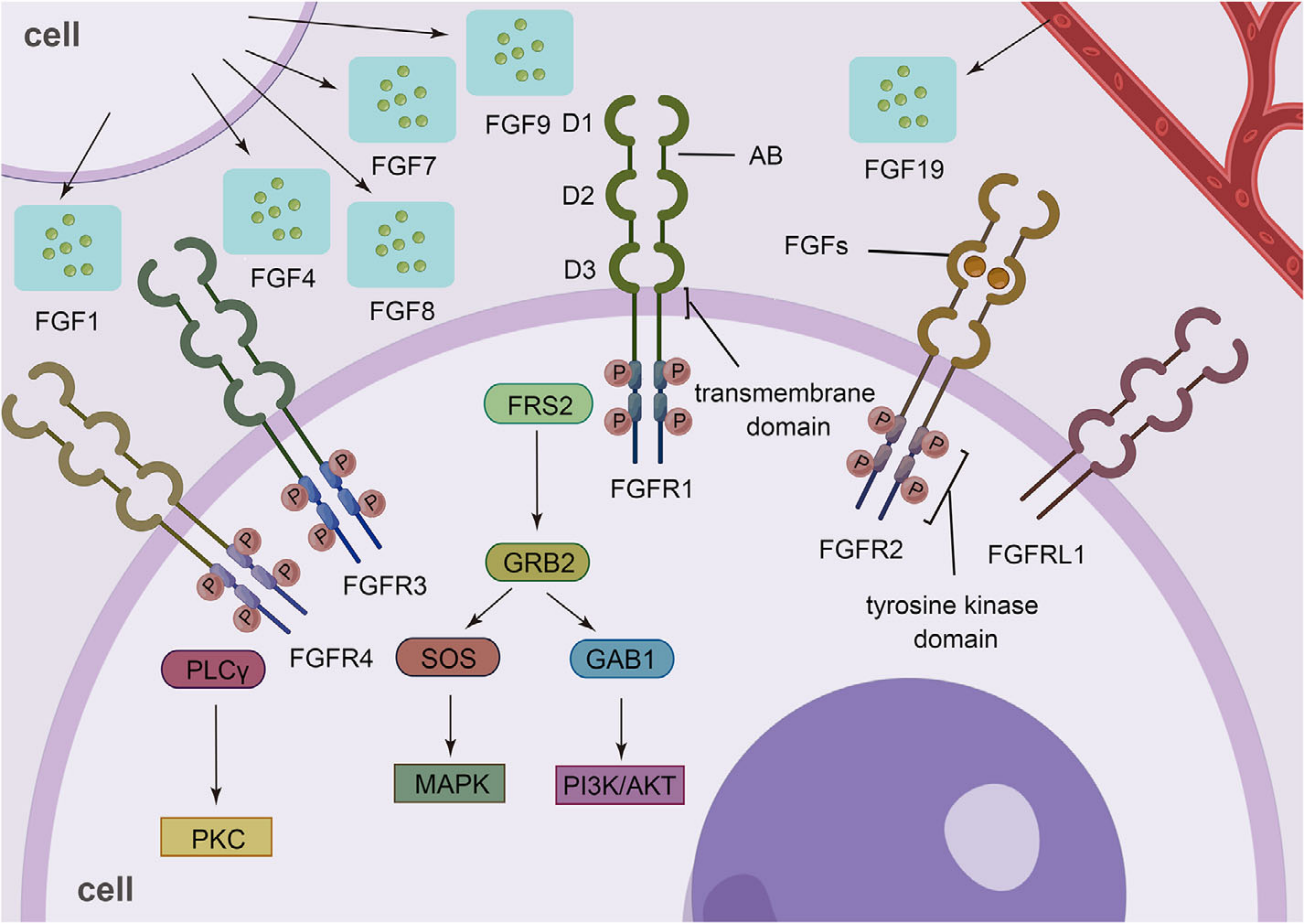
Basic structure of the FGFR family. There are five members of the FGFR family, FGFR1‐FGFR5, which all have an extracellular domain consisting of three immunoglobulin‐like domains (D1, D2, and D3), and a single transmembrane domain, differing in that FGFR5 lacks the intracellular tyrosine kinase domain.

There is a unique member of the FGFR family, known as FGFR like 1 (FGFRL1) or FGFR5. The extracellular domain of FGFRL1 is similar to the conventional extracellular domain of FGFR1–4, but the intracellular domain lacks the functional tyrosine kinase domain and contains only a short tail with a unique histidine‐rich motif.^88^, ^89^, ^90^ FGFRL1 was initially thought to act as a decoy receptor to negatively regulate the canonical FGFR signal pathway.^91^, ^92^ However, later evidence has demonstrated that FGFRL1 also participates in the canonical FGFR pathway and exerts a number of functions, such as promoting cell differentiation. The expression levels of FGFRL1 are elevated together with FGFR2 and FGFR1 during the differentiation of mesenchymal cells into osteoblasts and adipocytes, respectively.^93^ In pancreatic β cells, FGFRL1 interacts with SHP‐1 to enhance the ERK1/2 signaling pathway.^94^ Moreover, FGFRL1 is induced by inflammation and acts as a coreceptor for FGFR1, promoting FGFR1‐induced survival.^95^ (Figure 1)

## 3 ROLE OF FGFR IN NORMAL PHYSIOLOGY

During embryonic development, FGFR promotes cell growth, cell proliferation, cell survival, cell migration, angiogenesis, and organ development. In adult cells, FGFR contributes to tissue repair, wound healing, and metabolism.^96^, ^97^, ^98^, ^99^, ^100^, ^101^

### 3.1 Effect of FGFR on embryonic development

The FGFR pathway plays an active role in the development of almost all tissues and organs. When FGFR in mouse embryonic stellate or myofibroblastic cells is activated by FGF10, β‐catenin is activated, thus promoting the survival of hepatoblast cells.^102^ Furthermore, FGFR is involved in the regulation of the anterior–posterior pattern of zeugopod in chicken limb development, and it plays a role in the early critical period of eye field formation.^103^, ^104^ FGFR2‐mediated reciprocal regulatory loops between FGF8 and FGF10 are essential for limb induction.^84^ FGFR2 is the major FGFR expressed in Wolffian duct epithelial cells and is essential for the maintenance of the Wolffian duct, which is a primordium of the male reproductive tract, ureter, and kidney collecting duct system.^105^ Development of the stomach is dependent on FGFR2b.^106^ FGFR1 contributes to proliferation of mouse hippocampal progenitor cells.^107^ The FGFR1 signaling pathway promotes mitosis, leading to proliferation of myoblast cells and delaying their differentiation.^108^, ^109^ It is worth noting that the role of FGFR in cell differentiation remains unclear, but it may involve various FGFR members in different tissues. It has been shown that FGFRs, especially the most expressed FGFR1 and FGFR2, and their downstream ERK signaling pathway have a negative effect on odontogenic differentiation.^110^ FGFR3 has a negative effect on cartilage differentiation, which may be due to the interference of FGFR3 with Indian hedgehog/parathyroid hormone‐related protein signaling. At the same time, the Wnt/Lrp6 pathway is also activated.^111^, ^112^, ^113^ In contrast, FGFR2 expression is increased during the differentiation of mesenchymal stromal cells to osteoblasts, while FGFR1 expression in the process of adipocyte differentiation is increased; both processes are accompanied by an increase in FGFRL1 levels.^93^ During mouse embryonic development, the FGFRIIIc isoform drives the differentiation of embryonic stem cells into the endoderm.^114^

N‐cadherin targets the FGFR to inhibit its ubiquitination and lysosomal degradation, leading to ERK1/2 phosphorylation, which is essential for the migration of neocortical projection neurons.^115^ FGFR also regulates the proliferation and migration of lymphatic vessel endothelial cells by promoting glycolysis.^116^ In Xenopus laevis, it has been found that FGFR2–4 promote the expression of Sema3a in the forebrain, while the expression of Slit1 is dependent on FGFR1; both are mediated by the PIK3/AKT pathway, which synergistically directs retinal ganglion cell axons from the mid‐mesencephalon to the parietal membrane, thus forming a functional neural circuit.^117^, ^118^, ^119^ FGFR1 is the dominant FGFR expressed in endothelial cells, which promotes angiogenesis through downstream ERK1/2, c‐Jun N‐terminal kinase, p38 MAPK, and focal adhesion kinase.^120^, ^121^, ^122^ Because endothelial cells also have the ability to proliferate in adults, the above processes of lymphangiogenesis or angiogenesis may also occur in adult cells and participate in processes, such as inflammation or wound healing.

### 3.2 Effect of FGFR on adult cells

In adult cells, the promotion of FGFR1 for neovascularization, tissue repair, and wound healing is achieved by activating the JAK2/STAT3 pathway.^43^ In addition, FGFR1 may also be involved in angiogenesis and plaque instability within inflammatory plaques in rabbit atherosclerosis.^123^ Bile acids are important mediators of liver regeneration, while FGFR, activated by FGF15, maintains bile acid homeostasis and is an important mediator of bile acids for liver regeneration.^124^

## 4 FGFR IS INVOLVED IN HUMAN TUMORS

It has been shown that FGFR genetic alterations are present in 7.1% of tumors, of which 66% are amplification, 26% are mutations, and 8% are rearrangements. Alterations in FGFR1 are the most common (3.5%) followed by FGFR3 (2.0%), FGFR2 (1.5%), and FGFR4 (0.5%).^125^ In addition, some epigenetic alterations may also affect the expression levels or function of FGFR, thus activating significant signaling pathways to promote tumor cell proliferation, invasion, migration, epithelial–mesenchymal transition (EMT), angiogenesis, metabolic changes, chemoradiotherapy resistance, and tumor cell stemness maintenance^126^, ^127^, ^128^, ^129^ (Table 1).

**TABLE 1:** FGFR genetic alterations are associated with tumor development.

FGFRs	Tumors	Major genetic alterations	Diagnostic or prognostic makers	References
FGFR1	Breast cancer	Amplification	+	133, 134
	Lung cancer		+	152, 153
	Ovarian cancer		‐	163
	Bladder cancer		+	59
	Renal cell carcinoma		‐	164
	Prostate cancer		‐	165
	Esophageal carcinoma		‐	167
	Gastric cancer		+	168
	Colorectal cancer		‐	169
	Pancreatic cancer		‐	63, 170
	Head and neck squamous cell carcinoma		‐	171
	Osteosarcoma		‐	172
	Acute myeloid leukemia	Fusions or rearrangements	‐	188
	8p11 myeloproliferative syndrome		‐	189, 190
	Stem cell leukemia/lymphoma syndrome		‐	191
	Gliomas	Mutations	+	193
FGFR2	Gastric cancer	Amplification	+	208
	Colorectal cancer		‐	210
	Intrahepatic cholangiocarcinoma	Fusions or rearrangements	+	196, 197
	Gliomas		‐	205, 206
	Endometrial cancer	Mutations	+	212
	Melanomas		‐	213
	Breast cancer	Gene polymorphisms	+	229, 230
	Prostate cancer	Altered expression of splice isoform	+	237
	Renal cell carcinoma		‐	242
	Bladder cancer		‐	244
FGFR3	Gliomas	Fusions or rearrangements	‐	266, 267
	Lung cancer		‐	268, 269
	Bladder cancer	Mutations	+	251, 252
	Hepatocellular carcinoma		‐	261
	Renal cell carcinoma		‐	262
	Colorectal cancer	Overexpression	+	274
FGFR4	Hepatocellular carcinoma	Overexpression	+	276, 277
	Gastric cancer		+	285, 286
	Colorectal cancer		‐	287, 288
	Breast cancer		‐	289, 290
	Thyroid cancer		‐	291
	Nasopharyngeal carcinoma		‐	292
FGFRL1	Small cell lung cancer	Overexpression	‐	298
	Oral squamous cell carcinoma		‐	299
	Ovarian carcinoma		+	300
	Prostate cancer		+	301

Gene amplification, mutations, fusions, or rearrangements of FGFRs and unknown upregulation of FGFRs expression are associated with the development and progression of many tumors. The table shows the main genetic alterations of FGFR1–4 in various tumors, where “+” indicates that the genetic alteration can be used as a diagnostic or prognostic marker for the tumor, and “‐” indicates that it cannot or is not clear.

### 4.1 Regulatory network of FGFR1

FGFR1 is located at 8p11.23 and has a total length of 68,620 bases. FGFR1 is expressed in all tissues and organs throughout the body, especially in plasma, heart, and cerebrospinal fluid. FGFR1 is the most widely reported and studied gene among the FGFR family members, and it has been widely reported as an oncogene to promote tumor initiation and development,^130^ indicating its importance in tumorigenesis. The abnormal activation of FGFR1 is mainly associated with its genetic amplification, mutations, fusions, and rearrangements.

The most frequent abnormal activation of FGFR1 is gene amplification. The amplification rate of FGFR1 is 10−20% in estrogen receptor positive (ER+) breast cancer^131^, ^132^, ^133^ and 5% in triple negative breast cancer (TNBC).^134^ In TNBC, FGF2, as a ligand for FGFR1, activates the ERK/AKT pathway and promotes nuclear translocation of c‐Rel through FGFR1, which promotes the expression and secretion of S100A4. Paracrine activation of the S100A4/receptor for advanced glycation end products (RAGE) pathway promotes angiogenic effects in vascular endothelial cells and migration of tumor‐associated fibroblasts.^135^ Activation of the ERK/AKT pathway also phosphorylates or reprograms the ER, leading to chemoresistance to fulvestrant and the cyclin‐dependent kinase (CDK)4/6 inhibitor, palbociclib, in ER+ breast cancer.^136^, ^137^ Moreover, resistance to metformin of ER+ breast cancer cells is caused by FGFR1‐activated insulin receptor substrate 1/ERK signaling.^138^ Activation of the ERK2/c‐Fos/forkhead box Q1 (FOXQ1) pathway plays a key role in the growth of breast cancer cells.^139^ FGFR1 has been reported to enhance the expression of vascular endothelial growth factor A through tumor necrosis factor alpha‐induced protein 3, thus contributing to breast cancer angiogenesis.^140^ By gene set enrichment analysis, Riaz et al.^141^ found that FGFR1 is strongly correlated with GLI1 family zinc finger 1 (GLI1), a member of the Sonic hedgehog (SHH) pathway, and that the FGFR/GLI1 axis significantly promotes the metastasis of tumor cells. Furthermore, nuclear FGFR1, interacting with RNA‐Polymerase II (Pol II) and forkhead box protein A1 (FOXA1), regulates the transcription of target genes, which is a process independent of tyrosine kinase activity and mediates resistance of ER+ breast cancer cells to estrogen inhibitors and fulvestrant.^142^ Some noncoding RNAs regulate FGFR1 expression. For example, microRNA (miR)−338‐3p directly targets the 3′‐untranslated region of FGFR1 to reduce its levels, while circular RNA (circRNA)‐TFF1 acts as a sponge for miRNA‐338‐3p, indirectly restoring the expression of FGFR1.^143^ MiR‐133b inhibits breast cancer cell growth and resistance to cisplatin by targeting FGFR1 to inactivate the Wnt/β‐catenin pathway.^144^ MiR‐136 and miR‐326 directly bind to FGFR1, and circ‐0000518 competitively inhibits miR‐326.^145^, ^146^ Using bioinformatics, Boothby‐Shoemaker et al.^147^ found that leptin mRNA is positively correlated with FGFR1 mRNA in breast cancer. Subsequently, Pang et al.^148^ demonstrated that leptin‐induced pre‐B‐cell leukemia transcription factor 3 binds to the promoter of FGFR1, thereby activating the FGFR1 pathway and leading to letrozole resistance in tumor cells. The negative regulator of FGFR1, namely, circFGFR1‐encoded circFGFR1p, inhibits tumor cell development due to the absence of the tyrosine kinase structural domain.^149^

FGFR1 amplification is in 10−20% of patients with NSCLC^150^, ^151^, ^152^ and 5−7% of patients with small cell lung cancer.^153^, ^154^, ^155^ In KRAS mutant NSCLC, FGFR1 activates the MAPK/mTOR pathway, leading to increased expression of D‐cyclins and CDK6, resulting in resistance to palbociclib. In this case, the activation of mTOR is independent of AKT and is due to ERK phosphorylation of its key regulator, namely, tuberous sclerosis complex 2.^156^ Activation of the MAPK pathway has also been reported to lead to resistance to EGFR inhibitors and EMT of lung cancer cells, which was induced by hypoxia.^157^ Moreover, this drug resistance is also induced by activation of the AKT pathway.^158^ ERK downregulates beclin‐1 to inhibit autophagy of tumor cells.^159^ CircFGFR1 acts as a sponge for miR‐381‐3p to upregulate C‐X‐C motif chemokine receptor 4, thereby promoting the progression of NSCLC and resistance to anti‐PD‐1‐based therapy.^160^ In addition, miR‐22 in vascular endothelial cells in NSCLC tissues directly targets sirtuin 1 and FGFR1, inactivating their common AKT/mTOR pathway to inhibit angiogenesis and tumor cell growth.^161^ In small cell lung cancer, the promotion of tumor development by FGFR1 is mediated by the phospholipase C gamma 1 pathway, and retinoblastoma‐like protein 2 is negatively correlated with the expression of FGFR1.^162^

FGFR1 amplification also plays a role in ovarian cancer,^163^ bladder cancer,^59^ renal cell carcinoma,^164^ prostate cancer,^165^, ^166^ esophageal carcinoma,^167^ gastric cancer,^168^ colorectal cancer,^169^ pancreatic cancer,^63^, ^170^ head and neck squamous cell carcinoma,^62^, ^171^ and osteosarcoma.^172^ L1 cell adhesion molecule has been demonstrated to facilitate ovarian cancer cell spheroid formation, tumor initiation, and chemoresistance by triggering FGFR1 and its downstream SRC/STAT3 pathway.^173^ Glycosyltransferase 8 domain containing 2 induces resistance to Cis‐dichlorodiammine‐platinum treatment in ovarian cancer cells through activation of the FGFR/PI3K/AKT signaling pathway.^174^ In bladder cancer, tweety family member 3 inhibits phosphorylation of FGFR1 and downregulates the H‐Ras/A‐Raf/MEK/ERK signaling pathway (downstream targets include c‐Jun and c‐Fos) to inhibit the invasion and migration of bladder cancer cells.^175^ In prostate cancer, yes‐associated protein (YAP)/T‐box transcription factor 5 activates FGFR1 to mediate resistance to MET inhibitors.^176^ FGFR1 has also been shown to be associated with downregulation of choline kinase α and dysregulated choline metabolism, which promotes the progression of prostate cancer.^177^ HOTAIR promotes migration and invasion of prostate cancer cells by competitively inhibiting miR‐520b, which indirectly increases the expression of FGFR1.^178^ MiR‐1205 directly targets FGFR1 to inhibit colony formation, metastasis, and resistance to cisplatin in gastric cancer cells, while circARVCF reverses this effect.^179^ MiR‐198 and miR‐497 have also been found to target FGFR1.^180^, ^181^ Furthermore, AGAP2‐AS1 is highly expressed in colon cancer tissues, and it promotes colon cancer cell proliferation, migration, invasion, and resistance to gemcitabine via sponging miR‐497 to regulate FGFR1.^182^ In pancreatic cancer, SNHG1 also competitively inhibits miR‐497.^183^ The FGFR1/SRC/NF‐κB signaling axis plays a critical role in maintaining the stemness of pancreatic cancer cells.^184^ Interestingly, the nuclear translocation of FGFR1 and FGF2 in pancreatic stellate cells favors pancreatic cancer cell invasion.^185^ MiR‐573 also directly interacts with FGFR1 and reduces its expression levels, thereby inhibiting tumor cell migration, invasion, and EMT.^186^

**FIGURE 2:**
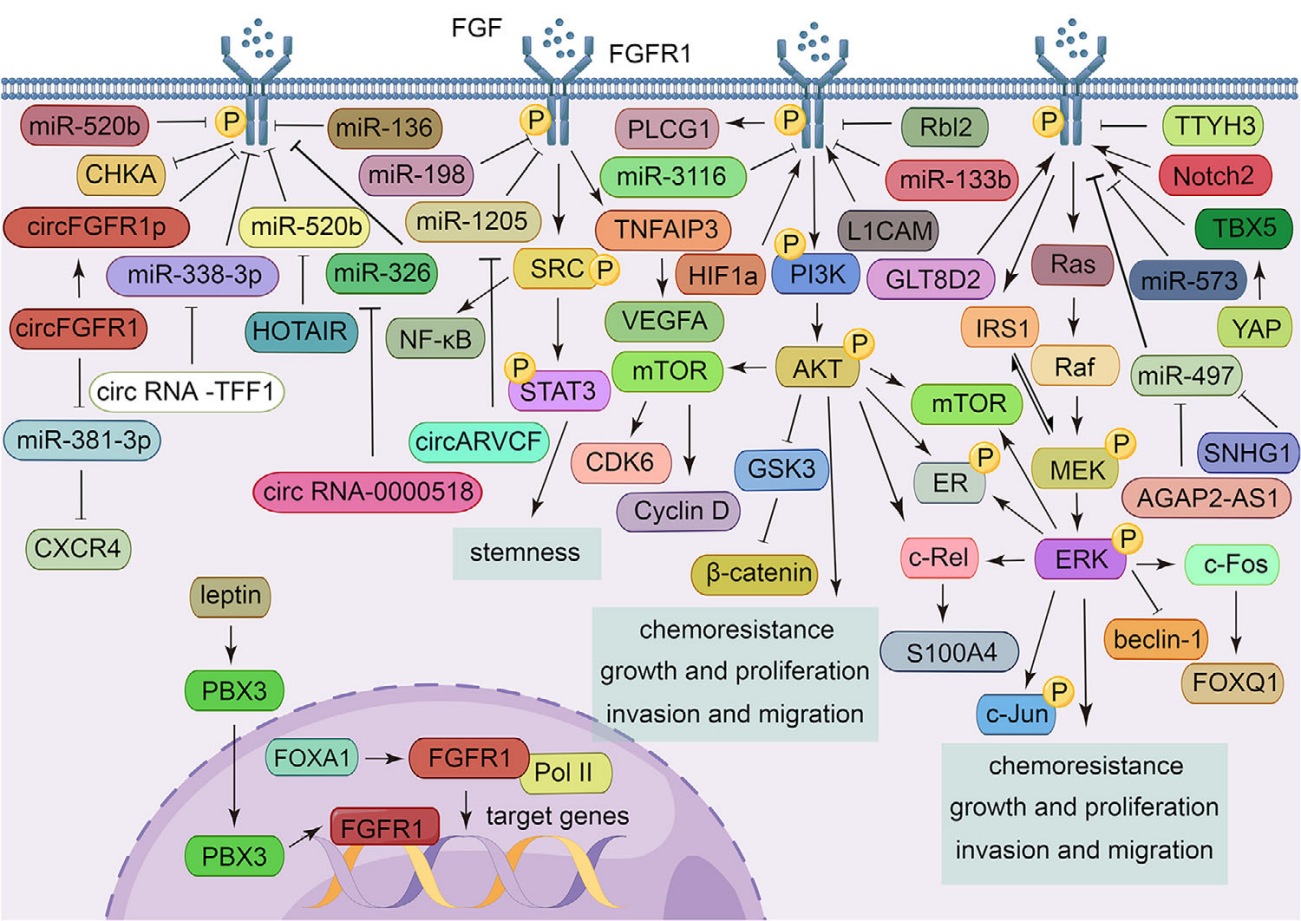
FGFR1 regulatory networks. In the cytoplasm, FGFR1 exerts its cancer‐promoting effects, mainly by activating the PI3K/AKT, Ras/ERK, and STAT pathways, to promote tumor cell proliferation, invasion, and metastasis as well as maintain tumor cell stemness and improve resistance to chemotherapy. In the nucleus, FGFR exerts its cancer‐promoting effects by targeting genes in a process independent of tyrosine kinase activity.

Although occurring infrequently, the gene mutations, fusions, or rearrangements of FGFR1 also lead to the development of some tumors. For example, FGFR gene fusions or rearrangements have been reported in squamous NSCLC,^187^ acute myeloid leukemia,^188^ 8p11 myeloproliferative syndrome,^189^, ^190^ and stem cell leukemia/lymphoma syndrome.^191^ The common fusion partners of NSCLC, acute myeloid leukemia, and 8p11 myeloproliferative syndrome are transforming acidic coiled‐coil‐containing protein 1 (TACC1), FGFR1 oncogene partner 2, and breakpoint cluster region, respectively. The mutation rate of FGFR1 p.N546K or p.K656E in low‐grade gliomas and mixed neuronal‐glial tumors is 6%, which is the fifth most common gene alteration in these tumors.^192^ In glioblastoma multiforme, Notch2 enhances the activity of FGFR1, thereby enhancing the AKT/glycogen synthase kinase 3 (GSK3) pathway to inhibit apoptosis.^193^ Notably, miR‐3116 inactivates the PI3K/AKT pathway by downregulating FGFR1, thus sensitizing glioma cells to temozolomide^194^ (Figure 2).

### 4.2 Role and signal network of FGFR2 in tumors

FGFR2 is located at 10q26.13 and has a length of 120,129 bases, and it is mainly expressed in the brain and spinal cord. Gene fusions or rearrangements of FGFR2 are the most common FGFR gene alteration events, and these are found almost exclusively in intrahepatic cholangiocarcinoma to facilitate its progress.^195^

In patients with intrahepatic cholangiocarcinoma, the incidence of FGFR2 rearrangements or fusions is 9−16%, and this is often accompanied by mutational inactivation of tumor protein P53, cyclin‐dependent kinase inhibitor 2A (CDKN2A), or BRCA1 associated protein‐1.^196^, ^197^, ^198^, ^199^ FGFR2 fusions usually involve exons 1−17, and the common fusion partners are BicC family RNA‐binding protein 1 (BICC1), KIAA1217, TACC2, coiled‐coil domain‐containing protein 6 (CCDC6), and adenosylhomocysteinase like 1. The FGFR2‐N/A fusion is when intron 17 or exon 18 of FGFR2 is fused to an intergenic region.^200^ The domain of FGFR2 is replaced by various fusion partners, and fusion partners then dimerize adjacent to FGFR2 to activate the tyrosine kinase domain along with a series of downstream signaling pathways.^201^ It has been reported that 32.9% of patients have a unique fusion partner, while 15.7% of patients have a fusion partner shared with only one other patient.^200^ This fusion diversity has been suggested to result in different biological effects.^50^ In addition, FGFR2 extracellular domain in‐frame deletions and the truncation of exon 18 also activate the downstream pathways of FGFR2.^201^, ^202^ SOX9, which is stimulated by the Wnt/β‐catenin pathway, enhances the transcription and expression of FGF7 and FGFR2, thereby stimulating the downstream pathway of FGFR2 to promote the proliferation of cholangiocarcinoma cells and resistance to pemigatinib.^203^ Through activation of the AKT/mTOR pathway, FGFR2 reduces the sensitivity of cholangiocarcinoma to gemcitabine.^204^ The correct folding of FGFR2–TACC3, FGFR2‐meningioma‐expressed antigen 5, and FGFR2–BICC1 requires the help of heat shock protein 90 (HSP90).^205^

FGFR2 fusions and rearrangements have also been found in gliomas.^205^, ^206^ In gliomas, FGFR2 phosphorylates phosphatase and tensin homolog on tyrosine 240, which interacts with Ki‐67 when exposed to ionizing radiation, resulting in a rapid increase and binding to chromosomes, thereby promoting the recruitment of RAD51 to facilitate DNA repair, ultimately leading to radiation resistance.^207^

FGFR2 is also subjected to gene amplification or mutations in gastric cancer,^208^, ^209^ colorectal cancer,^210^, ^211^ endometrial cancer,^171^, ^212^ and melanomas.^213^ FGFR2 promotes gastric cancer progression by downregulating thrombospondin‐4 (TSP4) through the PI3K/AKT/mTOR signaling axis.^214^ Similarly, FGFR2 activates the PI3K/AKT/mTOR axis and its downstream TSP1, which regulates migration and invasion of gastric cancer cells.^215^ FGF18, a ligand for FGFR2, interacts with FGFR2, enhances F‐actin, and then promotes nuclear aggregation of YAP1; FGFR2 also activates the MAPK pathway and its downstream molecule, c‐Jun, which upregulates YAP1 transcription to promote the progression of gastric cancer.^216^ Furthermore, some molecules act by regulating the levels of FGFR2 in gastric cancer. For example, there is a mutual positive regulation between CD44 and FGFR2.^217^ The posttranscriptional process of FGFR2 is regulated by the human DEAD/H‐box RNA helicase, DDX6, a protein encoded by a fusion gene.^218^ MiR‐381‐3p, miR‐494, miR‐5701, and miR‐519e‐5p also directly target FGFR2 to inhibit its expression and gastric cancer progression, while methyl‐CpG‐binding domain protein 1 and histone deacetylase 3 bind to form a complex that inhibits miR‐5701 expression, thereby restoring FGFR2 levels.^219^, ^220^, ^221^ Long noncoding RNA (lncRNA) ASNR also competitively inhibits miR‐519e‐5p.^222^ FGFR2 has been reported to mediate immune tolerance in colorectal cancer cells by inducing PD‐L1 expression through the JAK/STAT3 pathway.^223^ In endometrial cancer, FGFR2 has been reported to be activated in an autocrine manner, which activates AKT signaling and its downstream hairy and enhancer of split‐1 (HES1), thereby promoting tumor cell proliferation.^224^ Moreover, activating mutations in FGFR2 also induce Golgi fragmentation, loss of polarity, and directional migration.^225^

In addition, gene polymorphisms of FGFR2, including rs2981582,^226^, ^227^, ^228^, ^229^ rs1219648,^230^, ^231^ rs35054928, and rs45631563,^232^ have been found to be associated with susceptibility to breast cancer. In breast cancer, FGFR2 activates multiple pathways. Activation of the FGFR2/STAT3 pathway promotes proliferation, invasion, migration, and EMT of tumor cells.^233^ Ribosomal s6 kinase 2 interacts with FGFR2 to form an indirect complex that promotes FGF2‐triggered internalization of FGFR2.^234^ FGFR2 positively regulates PD‐L1 and helps tumor cells undergo immune escape.^235^ CD151 downregulates the posttranscriptional levels of FGFR2 via PKC, a process that requires HuR, a multifunctional RNA‐binding protein, and the assembly of processing bodies (P‐bodies).^236^

**FIGURE 3:**
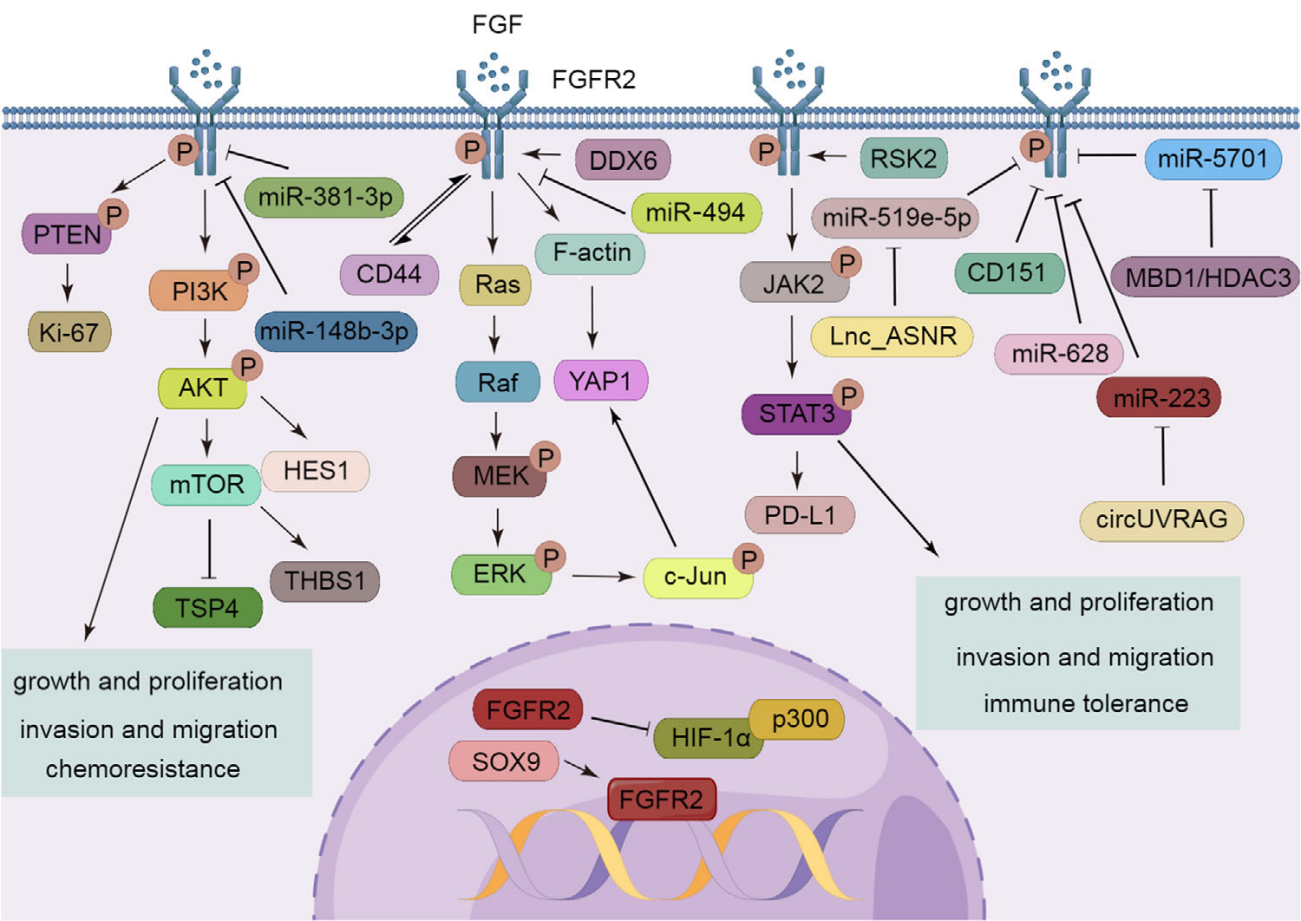
FGFR2 regulatory networks. In the cytoplasm, FGFR2 promotes tumor cell growth, tumor cell migration, and immune resistance through activation of the PI3K/AKT, Ras/ERK, and STAT pathways. In the nucleus, FGFR2 exerts tumor suppressive effects by inhibiting the recruitment of P300.

In some prostate cancers, the FGFR2 splice isoform, FGFR2 IIIc, has been found to be overexpressed, while FGFR2 IIIb expression is reduced.^237^, ^238^ FGFR2IIIb reverses the EMT of tumor cells and enhances sensitivity to docetaxel.^239^ MiR‐628 directly targets FGFR2 to inhibit the proliferation and invasion of prostate cancer cells.^240^ In contrast, Lee et al.^241^ suggested that nuclear FGFR2 plays an anticancer role by interacting with the transactivation domain of hypoxia‐inducible factor 1 (HIF1α) under hypoxic conditions, blocking the recruitment of coactivator p300 and thus leading to transcriptional repression of HIF target genes, which may be related to different subcellular localizations of FGFR. Conversion of FGFR2IIIb to FGFR2IIIc has also been identified in renal cell carcinoma and bladder cancer.^242^, ^243^, ^244^ MiR‐148b‐3p inhibits the growth and angiogenesis of renal cell carcinoma upon binding to FGFR2.^245^ Similarly, miR‐223 directly targets FGFR2 to promote bladder cancer growth and metastasis, an event that is counteracted by circUVRAG^246^ (Figure 3).

### 4.3 FGFR3 functions as a promoter in tumors

FGFR3 is located at 4p16.3 and contains 15,580 bases. FGFR3 is expressed in the brain, kidney, and testis, but it has low or no expression in the spleen, heart, and muscle. Abnormal activation of FGFR3 has been recognized to play a cancer‐promoting role in patients with urothelial cancer, especially bladder cancer, in which FGFR3 mutations, rearrangements, and fusions are the most common.^247^, ^248^ In addition, the tyrosine kinase inhibitor (TKI) has been shown to have a tangible effect, and its use has been approved in urothelial patients with FGFR abnormalities.^249^, ^250^

Bladder cancer is the most frequently occurring tumor with FGFR3 mutations, with an incidence of 9−11%.^251^, ^252^, ^253^ In bladder cancer, activation of the SRC pathway by FGFR3 leads to resistance of tumor cells to FGFR inhibitors, such as infigratinib or erdafitinib.^254^ FGFR3 upregulates ETS translocation variant 5 (ETV5) via the MAPK/ERK pathway, leading to elevated transcriptional coactivator with PDZ‐binding motif (TAZ), a cotranscriptional regulator of the Hippo signaling pathway involved in cell contact inhibition, which promotes the proliferation of bladder cancer cells.^255^ Moreover, activation of the FGFR3/RAS/MAPK pathway has also been found to promote the invasion of upper tract urothelial cancer.^256^ FGFR3 phosphorylates and activates the E3 ubiquitin ligase, neuronal precursor cell expressed developmentally downregulated 4, thereby inducing ubiquitinated degradation of PD‐L1 to regulate CD8^+^ T cell‐mediated immune detection.^257^ Acrolein from cigarette smoke induces cisplatin resistance in muscle invasive bladder cancer via the FGFR pathway.^258^ MiR‐181a‐5p directly targets FGFR3 to inhibit its expression and downstream STAT3 pathway, while circ_0068871 reverses this condition.^259^ MiR‐99a‐5p also directly binds to FGFR3 to inhibit mTOR signaling.^260^

Mutations in FGFR3 are also present in hepatocellular carcinoma (HCC)^261^ and renal cell carcinoma.^262^ FGFR3 regulates angiogenesis and metastasis in HCC by increasing the level of monocyte chemotactic protein 1.^263^ In addition, the FGFR3 splice isoform, FGFR3 IIIb/IIIc, and its ligand, FGF9, have been found to be upregulated in HCC, promoting tumor cell growth and aggressiveness.^264^ Similarly, a newly identified splicing mutant, FGFR3Δ7–9, which directly connects exon 6 and exon 10, directly phosphorylates the ten‐eleven translocation‐2 DNA demethylase and causes its ubiquitinated degradation, activating the AKT pathway to promote the proliferation of HCC.^125^, ^265^

FGFR3 may also undergo rearrangements or amplifications in gliomas^266^, ^267^ and lung cancer.^268^, ^269^ In glioma, the most common fusion partner of FGFR3 is TACC3, and the fusion process of FGFR3–TACC3, which requires HSP90, requires the involvement of cell division cycle 37 (CDC37).^270^ Furthermore, lncRNA CCAT1 promotes the proliferation, migration, and EMT of glioma cells through sponging miR‐181b.^271^ MiR‐99b targets FGFR3 to inhibit lung cancer progression.^272^ Interestingly, circFGFR3 has been reported to enhance galectin‐1 expression by competitively binding to miR‐22‐3p, thereby activating the AKT and ERK pathways to promote the proliferation and invasion of NSCLC cells.^273^

**FIGURE 4:**
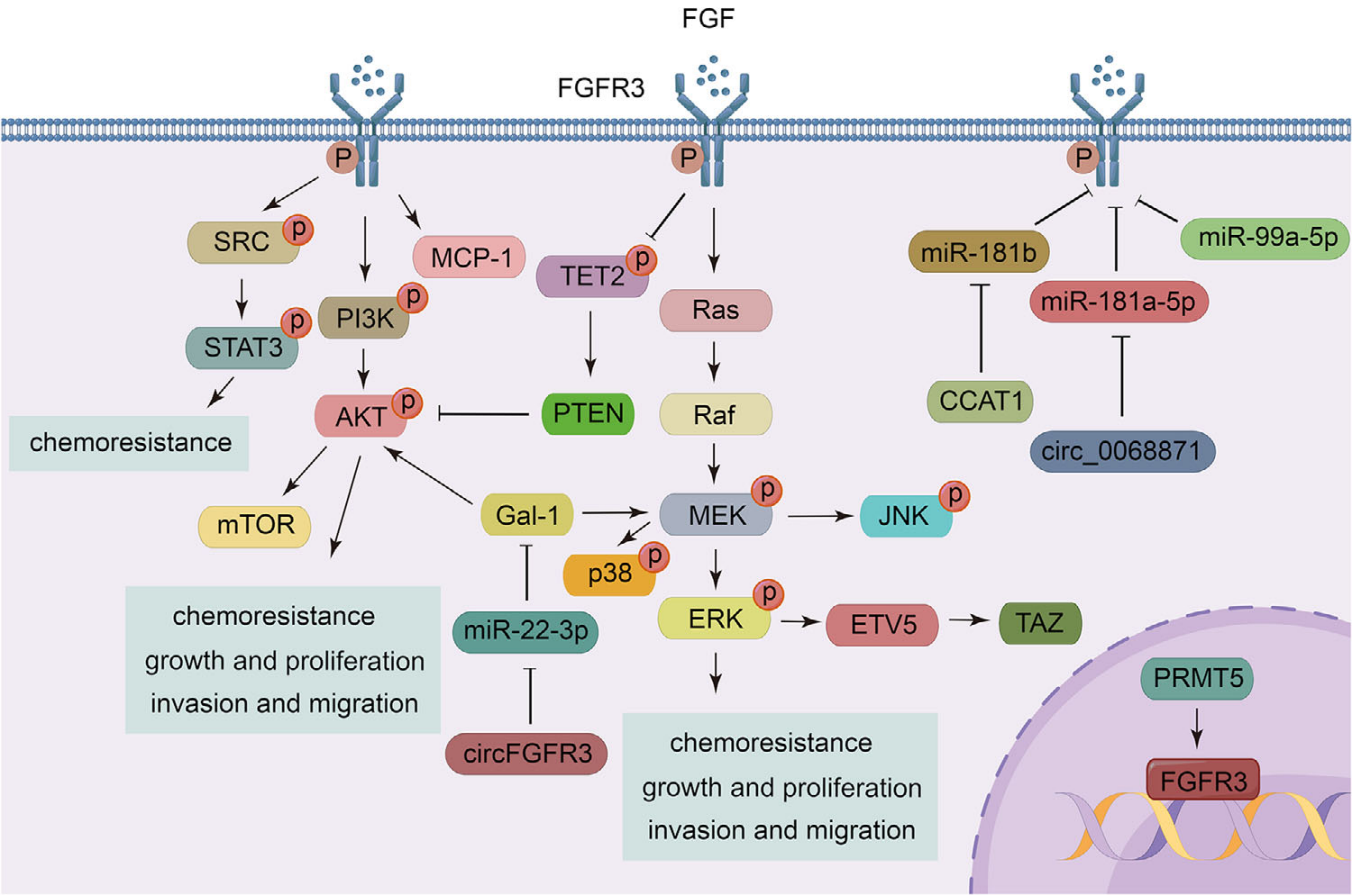
FGFR3 regulatory networks. FGFR3 promotes tumor cell growth, tumor cell migration, and chemoresistance through activation of the PI3K/AKT, Ras/ERK, and STAT pathways, and FGFR3 is regulated by various molecules, including numerous miRNAs.

Notably, FGFR3 is overexpressed in colorectal cancer, but this overexpression is not associated with genetic alterations but may be due to epigenetic modifications.^274^ In addition, protein arginine methyltransferase 5 methylates histone 4 to promote the transcription of FGFR3^275^ (Figure 4).

### 4.4 Oncogenic role and signal modulation of FGFR4

FGFR4 is located at 5q35.2 and has 11,230 bases, and it is mainly expressed in the lung and liver. As low probability events, the genetic alterations of FGFR4 have not been thoroughly investigated, but FGFR4 and its ligand, FGF19, have been found to be overexpressed and induce tumor development in many tumors, especially in HCC.^276^, ^277^, ^278^

Shin et al.^279^ found that FGFR4 activates the SRC/STAT3 pathway, and FGFR4 forms an endosomal complex with SRC and STAT3, which enters the nucleus in HCC, representing a potential mechanism of action for the FGFR4 pathway complex. Moreover, endoplasmic reticulum stress induces FGF19, which in turn activates GSK3β/Nrf2 signaling via FGFR4 to resist this event.^280^ Activation of GSK3β/β‐catenin signaling is also associated with EMT.^281^ FGFR4 activates store‐operated Ca2+ entry and its downstream nuclear factor of activated T cells‐c2 via PLCγ or the ERK pathway to promote self‐renewal of HCC stem cells.^282^ FGFR4 activates the PI3K/AKT/HIF1α pathway to promote transcription of homeobox B5, which in turn upregulates FGFR4, thereby promoting tumor cell metastasis.^283^ Furthermore, miR‐486‐3p directly targets FGFR4 to mediate growth inhibition and overcome sorafenib resistance in HCC.^284^

**FIGURE 5:**
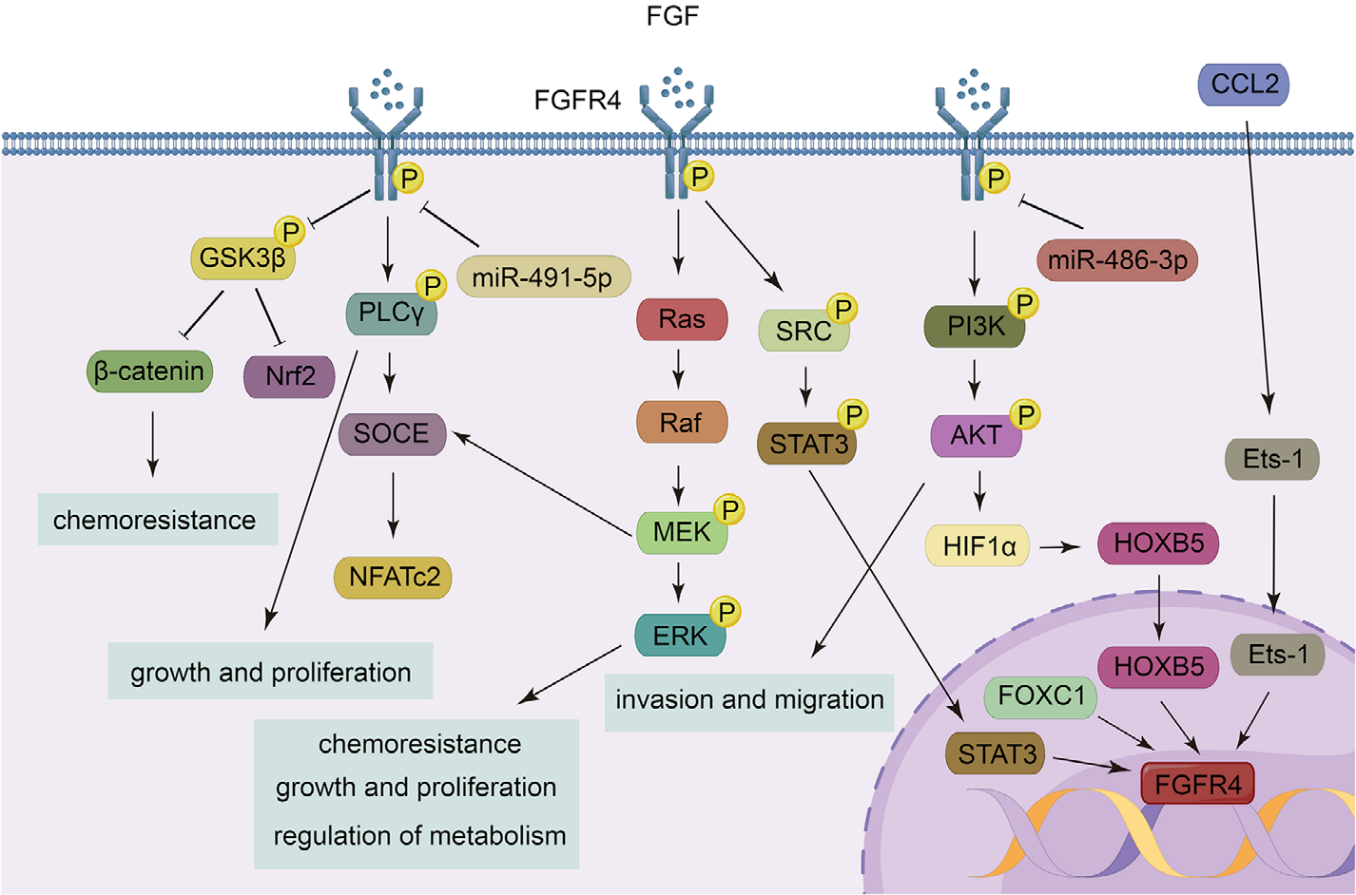
FGFR4 regulatory networks. Through activation of the PI3K/AKT, Ras/ERK, STAT, PLCγ, and Wnt/β‐catenin pathways, FGFR4 promotes the progression of a variety of tumors, while its expression levels are regulated by many molecules via transcriptional or posttranscriptional processes.

Overexpression of FGFR4 also occurs in gastric cancer,^285^, ^286^ colorectal cancer,^287^, ^288^ breast cancer,^289^, ^290^ thyroid cancer,^291^ and nasopharyngeal carcinoma.^292^ Helicobacter pylori infection increases the expression of FGFR4 in gastric cancer cells through activation of the STAT3 pathway by FGF19, and STAT3 binds directly to the FGFR4 promoter, forming a feed‐forward response loop.^293^ In addition, miR‐491‐5p indirectly downregulates FGFR4 levels.^294^ In colon cancer tissues, tumor‐associated fibroblasts generate chemokine ligand 2, which acts on its receptor on the tumor surface to enhance the transcription of FGFR4 in an Ets‐1‐dependent manner, thereby activating the β‐catenin pathway to lead to EMT.^295^ The expression of FGFR4 is also regulated by forkhead box C1 (FOXC1), which directly targets FGFR4, promotes its transcription, and drives metastasis of colorectal cancer cells.^287^ In human epidermal growth factor receptor 2 (HER2)+ breast cancer, epigenetic alteration of m6A hypomethylation causes FGFR4 to phosphorylate GSK3β and then activate the β‐catenin/transcription factor 4 signaling pathway, leading to anti‐HER2 therapeutics in tumor cells.^296^ Activation of the MAPK/ERK pathway leads to elevated glucose metabolism and resistance to adriamycin in breast cancer^297^ (Figure 5).

### 4.5 Action and molecular basis of FGFRL1 in tumors

**FIGURE 6:**
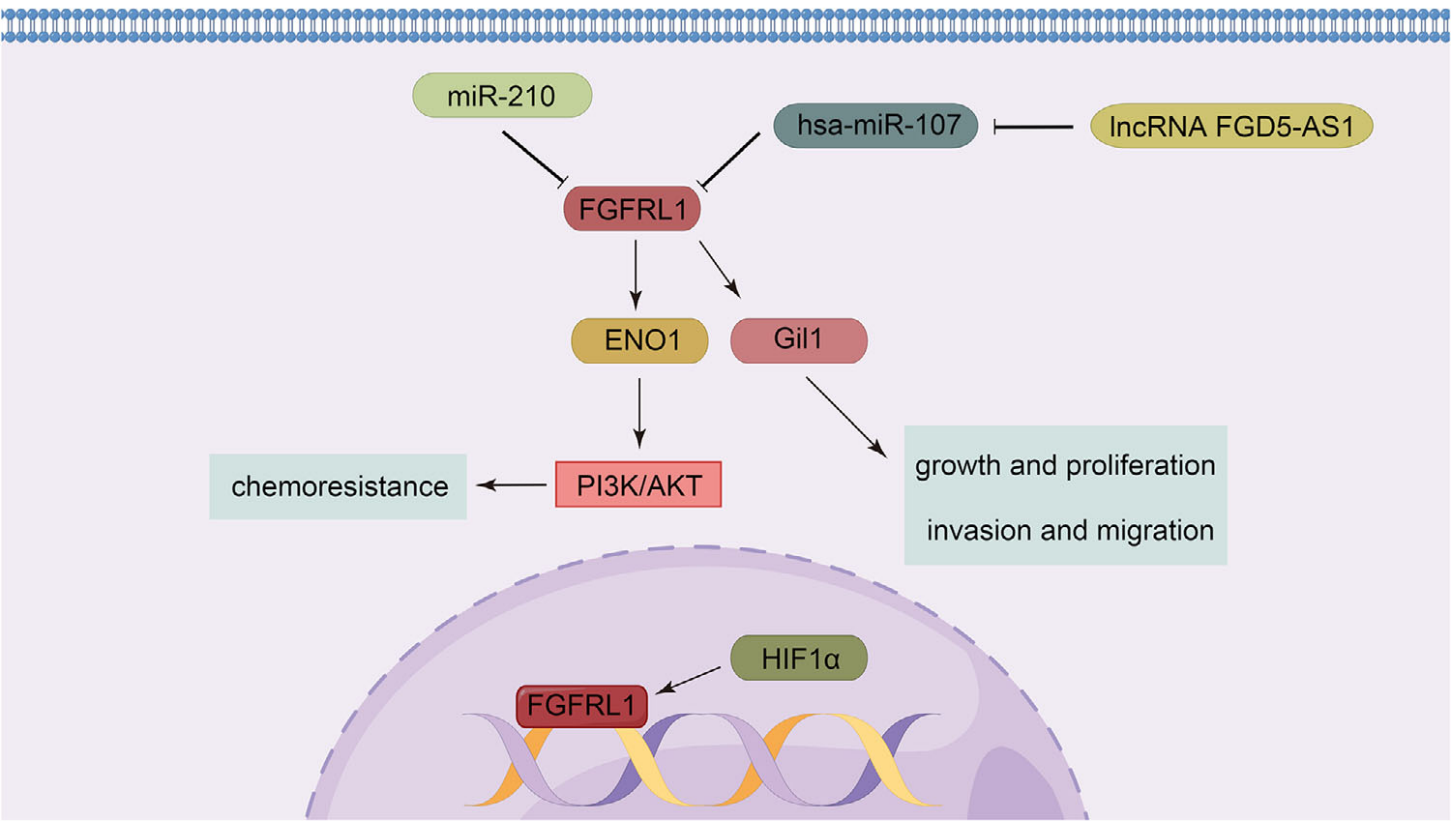
FGFRL1 regulatory networks. Despite the lack of an intracellular tyrosine kinase structural domain, FGFRL1 promotes tumor progression by interacting with various molecules and indirectly activating a number of pathways.

FGFRL1 is located at 4p16.3 with a length of 16,963 bases; it is preferentially expressed in cartilage tissue and pancreas, and weakly expressed in lung, small intestine and spleen. FGFRL1 has been reported to have elevated expression in small cell lung cancer,^298^ oral squamous cell carcinoma,^299^ ovarian carcinoma,^300^ and prostate cancer.^301^ Despite lacking the intracellular tyrosine kinase domain, FGFRL1 interacts with a number of proteins to indirectly activate several signaling pathways, thereby promoting tumor development. For instance, in small cell lung cancer, enhanced FGFRL1 interacts with alpha‐enolase to activate its downstream PI3K/AKT pathway, leading to chemotherapy resistance.^298^ Under hypoxic conditions, HIF1α binds to the promoter of FGFRL1 to promote it expression levels. Subsequently, FGFRL1 upregulates Gil1 and activates the Hedgehog (Hh) pathway, thereby promoting the growth, proliferation, and migration of ovarian cancer cells.^302^ Moreover, the expression of FGFRL1 is regulated by a number of molecules. In esophageal squamous cell carcinoma, miR‐210 directly targets FGFRL1 to induce cell death and cell cycle arrest in G1/G0 and G2/M, thereby inhibiting cancer cell survival and proliferation.^303^ Similarly, miR‐210 plays the same role in HCC.^304^ In NSCLC, lncRNA FGD5‐AS1 indirectly upregulates FGFRL1 levels and promotes cancer cell proliferation through sponging hsa‐miR‐107^305^ (Figure 6).

## 5 FGFR ACTS AS A THERAPEUTIC TARGET

FGFR amplification, mutations, rearrangements, or fusions are considered as potential biomarkers of FGFR therapeutic response. However, it is important to note that the amplification of FGFR is not equivalent to overexpression. There have been many findings indicating that the association between FGFR amplification and overexpression is not strong. For example, Gonzalez‐Ericsson et al.^131^ found that only 50% of FGFR‐amplified breast cancers exhibit protein overexpression. And, there is evidence that it is the high expression levels of FGFR1–4 rather than copy‐number alteration that is strongly associated with the efficacy of FGFR inhibitors.^306^ Similarly, Bogatyrova et al.^307^ suggested that the overexpression of FGFR1 in NSCLC is better explained by promoter demethylation or downregulation of specific miRNAs, which may be related to the poor effect of FGFR TKIs in some patients with FGFR amplification. The difference in FGFR amplification fragments may be the cause of the above events. Therefore, it may be better to use FGFR amplification and overexpression as a combined treatment indicator. In addition, a series of genetic alterations in FGFR2 have been found to result in transcription of exon 18‐truncated FGFR2, which should perhaps also be considered as an indicator of the effectiveness of FGFR‐targeted therapy.^308^

TKIs can be divided into nonselective and selective inhibitors. Nonselective inhibitors include ponatinib (AP24534), lenvatinib, ninetedanib (BIBF 1120), olverembatinib (GZD824, HQP1351), dovitinib (TKI258, CHIR‐258), lucitanib (AL3810), and derazantinib (ARQ 087). Selective inhibitors include futibatinib (TAS120), erdafitinib (JNJ‐42756493), LY2874455, pemigatinib (INCB054828, FIGHT‐101), infigratinib (BGJ398), AZD4547, rogaratinib (BAY1163877), E7090 (tasurgratinib), and debio 1347 (CH5183284) (Table 2).

**TABLE 2:** Common TKIs and the cancers they treat and their adverse effects.

Category	Drugs	Target points	Drug status	Main types of cancer treated	Side effects	clinical trial number	References
Non‐selective TKIs	Ponatinib	FGFR 1–4	Marketed	Philadelphia chromosome‐positive acute lymphoblastic leukemia, chronic myeloid leukemia	Pancreatitis, hypertension, hyperlipidemia, liver dysfunction, and AOE	‐	309, 310
	Lenvatinib			Hepatocellular carcinoma, thyroid cancer, renal cell carcinoma	Fatigue, loss of appetite, diarrhea, weight loss, hypertension, and some liver‐related side effects	‐	311, 312
	Nintedanib	FGFR 1–3		Non‐small cell lung cancer	Gastrointestinal reactions, decreased platelets and hypertension	‐	313, 314
	Olverembatinib			Chronic myeloid leukemia	Skin pigmentation, thrombocytopenia, hypocalcemia, proteinuria, and hyperuricemia	‐	315, 316
	Dovitinib		Phase 3 clinical trial	Breast cancer, hepatocellular carcinoma, renal cell carcinoma prostate cancer	Nausea, vomiting, fatigue, anorexia, and diarrhea	NCT02116803, NCT01223027	317, 318
	Lucitanib	FGFR1 and FGFR2		Small cell lung cancer, breast cancer, nasopharyngeal carcinoma	Hypertension, hypothyroidism, nausea, and proteinuria	NCT04254471	319, 320
	Derazantinib	FGFR 1–3	Phase 2 clinical trial	Intrahepatic cholangiocarcinoma	Fatigue, ocular toxicity, and hyperphosphatemia	NCT03230318, NCT05174650	321, 322
Selective TKIs	Futibatinib	FGFR 1–4	Marketed	Intrahepatic cholangiocarcinoma	Hyperphosphatemia, diarrhea, and nausea	‐	341, 342
	Erdafitinib			Uroepithelial carcinoma	Hyponatremia, stomatitis, hyperphosphatemia, and weakness	‐	343
	Pemigatinib	FGFR 1–3		Cholangiocarcinoma	Hyperphosphatemia, hypophosphatemia, arthralgia, stomatitis, hyponatremia, abdominal pain, fatigue, and fever	‐	195, 346
	Infigratinib			Cholangiocarcinoma	Hyperphosphatemia, fatigue, stomatitis, and alopecia	‐	347, 348
	AZD4547		Phase 3 clinical trial	Breast cancer, squamous cell lung cancer	Hyperphosphatemia, dry mouth, hair loss, taste disturbance, constipation, nausea, and retinal pigment epithelial detachment	NCT02965378	349, 350
	Rogaratinib			Uroepithelial carcinoma	Diarrhea, decreased appetite, fatigue, and asymptomatic lipase elevation	NCT03410693	351, 352
	E7090		Phase 2 clinical trial	Cholangiocarcinoma		NCT04238715, NCT04962867	353, 354
	Debio 1347			Breast cancer, cholangiocarcinoma	Hyperphosphatemia, diarrhea, constipation, fatigue, loss of appetite, and dry mouth	NCT03834220, NCT03344536	355, 356
	LY2874455	FGFR 1–4	Phase 1 clinical trial	Gastric cancer, head and neck squamous cell carcinoma	Hyperphosphatemia, diarrhea, and stomatitis	NCT01212107	344, 345

TKIs are usually classified as selective or nonselective, with different mechanisms of action, targets, and side effects.

### 5.1 TKIs

#### 5.1.1 Nonselective TKIs

Non‐selective inhibitors include ponatinib,^309^, ^310^ lenvatinib,^311^, ^312^ ninetedanib,^313^, ^314^ olverembatinib,^315^, ^316^ dovitinib,^317^, ^318^ lucitanib,^319^, ^320^ and derazantinib.^321^, ^322^ Nonselective inhibitors are characterized by their ability to inhibit FGFR as well as some other kinases, such as vascular endothelial growth factor receptor (VEGFR), platelet‐derived growth factor receptor (PDGFR), and fetal liver tyrosine kinase receptor (FLT).

Ponatinib has been shown to be active in a variety of tumors, such as philadelphia chromosome‐positive acute lymphoblastic leukaemia, chronic myeloid leukemia, endometrial cancer, bladder cancer, gastric cancer, breast cancer, lung cancer, and colon cancer, and it acts on more than 40 kinases, including FGFR, SFKs, VEGFR, and PDGFR.^323^, ^324^ Ponatinib has significant adverse effects, mainly including pancreatitis, hypertension, hyperlipidemia, liver dysfunction, and arterial occlusive events (AOEs).^325^ Lenvatinib is a first‐line systemic chemotherapeutic agent used in patients with unresectable HCC that inhibits FGFR1–4, thereby reducing tumor stem cells in HCC.^326^ It can also be used for thyroid cancer and renal cell carcinoma. Lenvatinib also targets VEGFR1–3, PDGFRα, RET, and KIT. The most common adverse effects of lenvatinib treatment include fatigue, loss of appetite, diarrhea, weight loss, hypertension, and some liver‐related side effects, such as ascites and hepatic encephalopathy.^327^ Nintedanib is approved in combination with docetaxel for the treatment of locally advanced, metastatic or locally recurrent non‐small cell lung cancer after chemotherapy due to its ability to block FGFR1–3, EGFR1–3, and PDGFFRα and β. Common adverse reactions include gastrointestinal reactions, decreased platelets and hypertension.^328^, ^329^ Olverembatinib binds to FGFR1–3, FLT3, and PDGFRα, and it overcomes the drug resistance activity of some mutations, such as FGFR1‐V561F/M, indicating that it is a new drug to target FGFR.^330^ In addition, as an approved drug, it can treat T315I‐mutated chronic myeloid leukemia.^315^, ^331^ Common adverse events with olverembatinib include skin pigmentation, thrombocytopenia, hypocalcemia, proteinuria, and hyperuricemia.^332^ The primary target of dovitinib is VEGFR1–3, but it also targets FGFR1–3, PDGFRβ, FMS‐like tyrosine kinase 3 (FLT3), KIT, RET, TrkA, and colony stimulating factor 1 (Csf‐1). Dovitinib has shown therapeutic effects in breast cancer, HCC, prostate cancer, renal cell carcinoma, melanoma, multiple myeloma, and gastrointestinal mesenchymal tumors and its major drug‐related toxicities include nausea, vomiting, fatigue, anorexia, and diarrhea.^333^, ^334^, ^335^ Lucitanib strongly inhibits FGFR1, FGFR2, and VEGFR1–3, and it has antiangiogenic and broad‐spectrum antitumor activity against several cancers, including small cell lung cancer, breast cancer, nasopharyngeal carcinoma, colorectal cancer, ovarian cancer, and kidney cancer.^336^, ^337^ Common adverse events of lucitanib include hypertension, hypothyroidism, nausea, and proteinuria.^338^ Derazantinib inhibits FGFR1–3, CSF1R ahd VEGFR2. It has shown antitumor activity in advanced, unresectable intrahepatic cholangiocarcinoma that has progressed after chemotherapy, and the treatment‐related adverse events of derazantinib include fatigue, ocular toxicity, and hyperphosphatemia.^339^, ^340^

As nonselective inhibitors, drug toxicity is a major problem that the above mentioned drugs have to face, but it is undeniable that some drugs have shown resistance to mutations, such as the above mentioned third generation TKIs olverembatinib.

#### 5.1.2 Selective TKIs

Selective inhibitors include futibatinib,^341^, ^342^ erdafitinib,^343^ LY2874455,^344^, ^345^ pemigatinib,^195^, ^346^ infigratinib,^347^, ^348^ AZD4547,^349^, ^350^ rogaratinib,^351^, ^352^ E7090,^353^, ^354^ and debio 1347.^355^, ^356^ Selective inhibitors target FGFR to inhibit downstream signaling pathways, thereby acting as cancer suppressors. Futibatinib,^357^, ^358^ erdafitinib,^359^, ^360^ and LY2874455 are pan‐FGFR inhibitors. Futibatinib, a non‐ATP‐competitive FGFR1–4 inhibitor that binds covalently and irreversibly to the conserved cysteine in the P‐loop of the FGFR kinase domain, has resulted in remission in various cancers, including cholangiocarcinoma, gastric cancer, uroepithelial cancer, central nervous system tumors, head and neck cancer, and breast cancer, with the greatest activity in intrahepatic cholangiocarcinoma with FGFR2 rearrangements or fusions. And, it has been approved in September 2022 for the treatment of adult, previously treated, unresectable, locally advanced or metastatic FGFR2 fusions or rearrangements intrahepatic cholangiocarcinoma patients. In addition, as a third‐generation irreversible TKI, futibatinib is able to overcome resistance caused by some mutations in the FGFR kinase domain, including FGFR2‐K660M, N550K, and L618V.^361^ The most common adverse events of futibatinib are hyperphosphatemia, diarrhea, and nausea.^362^ Erdafitinib has been approved for patients with uroepithelial carcinoma with abnormal FGFR; its common adverse effects include hyponatremia, stomatitis, hyperphosphatemia, and weakness.^363^ LY2874455 has been found to be useful in gastric and head and neck squamous cell carcinoma, and, notably, as a novel drug, it is resistant to most FGFR mutations resulting in drug resistance, including FGFR4 V550L and FGFR1–561 M, which partially compensates for the lack of futibatinib action, but its specific action needs to be further clarified. Its main side effects include hyperphosphatemia, diarrhea, and stomatitis.^364^, ^365^ The inhibitors of FGFR1–3 are pemigatinib,^366^ infigratinib, AZD4547,^367^ rogaratinib, E7090 and debio 1347. Pemigatinib and infigratinib have been approved for cholangiocarcinoma with FGFR fusions or rearrangements. The most common adverse effect of pemigatinib is hyperphosphatemia, and other adverse effects of pemigatinib include hypophosphatemia, arthralgia, stomatitis, hyponatremia, abdominal pain, fatigue, and fever.^195^ Adverse events associated with infigratinib include hyperphosphatemia, fatigue, stomatitis, and alopecia.^368^ AZD4547 has significant efficacy in breast cancer and squamous cell lung cancer patients, and the common adverse events of AZD4547 include hyperphosphatemia, dry mouth, hair loss, taste disturbance, constipation, nausea, and retinal pigment epithelial detachment.^369^ Rogaratinib has been shown to be effective in patients with uroepithelial and head and neck squamous cell carcinoma with overexpressed levels of FGFR. Common adverse effects include diarrhea, decreased appetite, fatigue, and asymptomatic lipase elevation.^370^, ^371^ E7090 selectively inhibits FGFR1–3 and has been found to be effective in patients with cholangiocarcinoma with FGFR2 gene fusions and in patients with gastric cancer with FGFR2 gene amplification or increased expression, but more information is needed for a larger sample size.^372^ Debio 1347 is an ATP‐competitive, highly selective FGFR1–3 inhibitor, and it is mainly used to treat breast cancer and cholangiocarcinoma. The common adverse effects of debio 1347 include hyperphosphatemia, diarrhea, constipation, fatigue, loss of appetite, and dry mouth.^373^

### 5.2 Non‐TKIs FGFR‐targeted drugs

**FIGURE 7:**
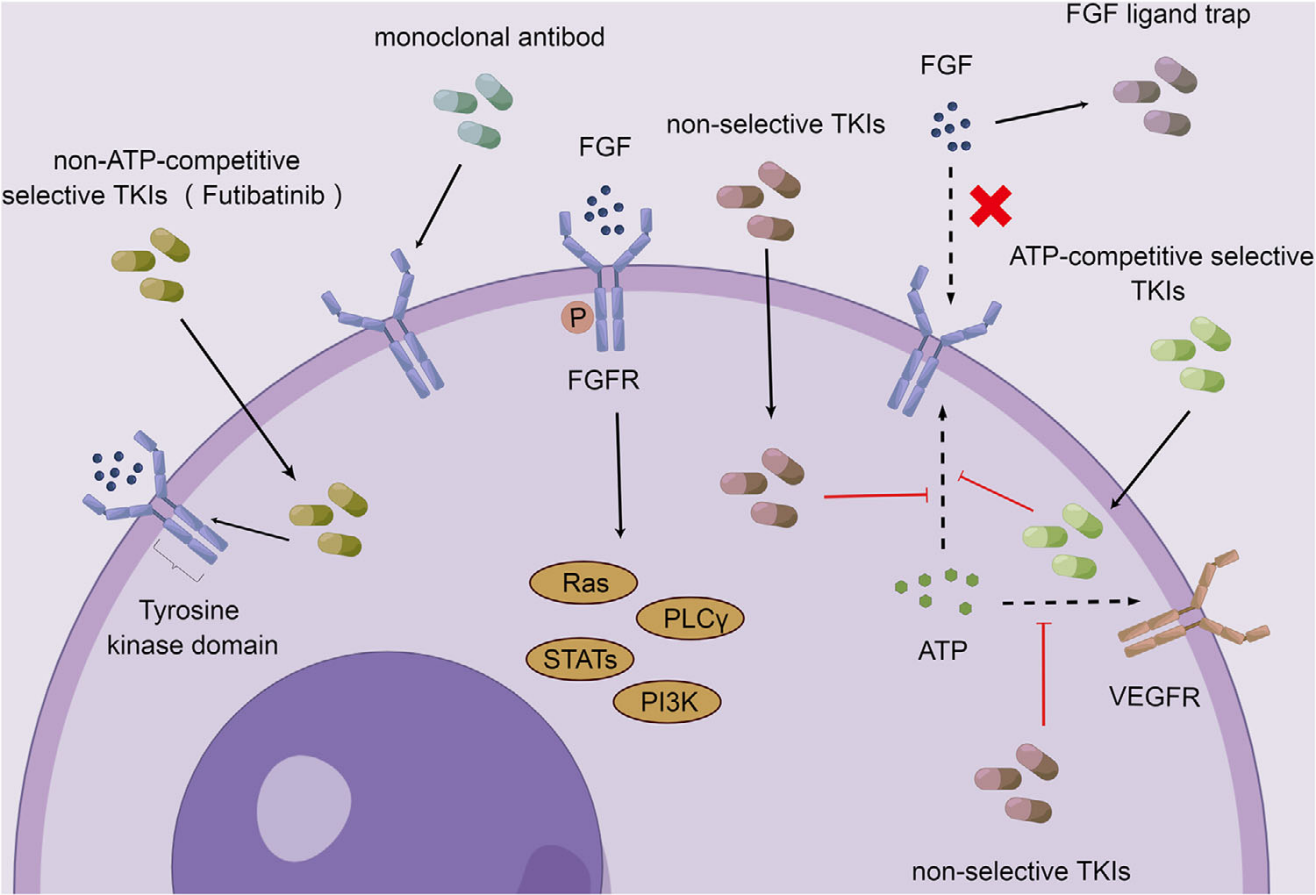
Major FGFR‐targeted drugs in tumors. The target drugs of FGFR in tumors are mainly TKIs, among which, futibatinib as a non‐ATP‐competitive inhibitor can directly bind to the P‐loop of kinase domain, while other drugs are ATP kinase inhibitors, in addition, there are also monoclonal antibody drugs and FGF ligand trap.

In addition to the TKIs mentioned above, here are some other drugs that target FGFR. For example, bemarituzumab (FPA144), a monoclonal antibody against FGFR2IIb, is still in clinical trials and is used to treat patients with FGFR2IIb overexpression, including gastroesophageal cancer. It reduces the risk of hyperphosphatemia compared with the TKIs mentioned above. Of course, it still has some side effects, such as neutropenia, corneal disease, and stomatitis.^374^, ^375^, ^376^ And there is the FGF ligand trap, FP‐1039 (GSK3052230), which is the extracellular ligand binding domain of FGFR1 fused to the Fc segment of human immunoglobulin G1, thereby inhibiting the activation of FGFR by FGF. Its common adverse effects include neutropenia, hair loss, nausea, joint pain, weakness and diarrhea^377^, ^378^, ^379^ (Figure 7).

## 6 SUMMARY AND OUTLOOK

In tumor tissues, some genetic or epigenetic changes in FGFR, such as gene amplification, mutations, fusions, or rearrangements, as well as histone methylation or noncoding RNAs mediate the elevation of FGFR expression, lead to aberrant activation of FGFR, which upregulates a number of unique pathways, such as the PI3K/AKT, Ras/ERK, PLCγ, STAT, and Wnt/β‐catenin signaling pathways, to promote tumorigenesis and progression, including tumor cell proliferation, tumor cell invasion, tumor cell migration, EMT, angiogenesis, metabolic changes, chemoradiotherapy resistance, and tumor cell stemness. Because auto‐phosphorylation of FGFR plays a key role in the activation of these pathways, TKIs have become an important strategy for inhibiting the action of FGFR.

Several drugs are now approved for use in cancer treatment. For example, erdafitinib is approved for patients with uroepithelial carcinoma with abnormal FGFR, and pemigatinib and infigratinib are approved for patients with cholangiocarcinoma with FGFR fusions or rearrangements.^362^ However, TKIs may have several problems throughout the treatment process. Among some patients with alterations in FGFR, especially FGFR amplification, TKIs are less effective. First, as mentioned above, FGFR amplification is not strongly associated with elevated FGFR expression in some cancers.^307^ Therefore, it remains unclear whether FGFR amplification and increased expression should be used as a combined TKIs treatment indicator. Second, the regulatory mechanism of nuclear FGFR on its target genes is independent of tyrosine kinase activity.^142^ On this point, the role of other non‐TKIs FGFR‐targeting agents needs to be further confirmed. Therefore, the exploration of new targets for FGFR is necessary. Third, because FGFR mutations are accompanied by changes in some other tumor promoter, tumor suppressor, or pathways, such as P53, EGFR, or MAPK signaling, combination therapy may be a new breakthrough point. There have been some studies.^380^, ^381^, ^382^ For example, the nonselective TKI lenvatinib is thought to inhibit FGFR with feedback activation of the EGFR–PAK2–ERK5 signaling axis, while the combination of lenvatinib and the EGFR inhibitor gefitinib has been found to be clinically meaningful in patients with advanced HCC.^311^ In KRAS‐mutated lung adenocarcinoma, the use of the MEK inhibitor trametinib leads to abnormal activation of FGFR1, which leads to the development of drug resistance. However, in combination with FGFR inhibitors, this resistance was suppressed.^383^ In addition, in a mouse model of lung cancer with p53mut, erdafitinib was found to promote T cell cloning and expansion and promote anti‐tumor immunity when combined with PD‐1 blockers.^384^ But the specific efficacy and toxic effects need to be studied in depth. In addition, some patients have developed resistance to TKIs. There are many mechanisms for the development of drug resistance, the most common of which are FGFR kinase and especially gatekeeper mutations. Gatekeeper residues are located in the hinge region of the ATP‐binding pocket of the kinase, and when they are mutated, they cause spatial inhibition of the ATP pocket, thus blocking entry of TKIs and causing drug resistance.^385^ Common gatekeeper mutations include FGFR1–V561F/M, FGFR2–V564F/I, V565I, and FGFR3–V555M.^362^, ^386^ In addition, the development of FGFR resistance is associated with the activation of some related pathways, that is, bypassing the inhibitory effect of TKIs on the FGF/FGFR signaling pathway, which may result from other mutations in nongatekeepers. For instance, FGFR2 N550H mutation was found to upregulate the PI3K/AKT/mTOR signaling pathway.^387^ Some new, mutation‐targeting drugs have emerged, such as futibatinib and GZD824, but they still have some limitations.^330^ In summary, there is a demand for the development of new FGFR inhibitory drugs as well as the identification of combinations of drugs (related pathway molecular inhibitors) and other covariant gene inhibitors.

## AUTHOR CONTRIBUTION

Q. L., J. Y. H., and W.W.Y. collected the related reports. Q. L. drafted the manuscript and discussed the concepts of the manuscript. J. Y. H. and W.W.Y. provided valuable discussion. Z. L., S. L., and W. Y. F., provided valuable discussion and revised the manuscript. W. Y. F. participated in designing the review. All authors read and approved the final manuscript.

## CONFLICT OF INTEREST STATEMENT

The authors declare no conflict of interest.

## ETHICS STATEMENT

Not applicable.

## Data Availability

Not applicable.
